# Staple Line Reinforcement during Sleeve Gastrectomy with SeamGuard: Single-Center Retrospective Case-Control Study over a 5-Year Period

**DOI:** 10.3390/jcm13123410

**Published:** 2024-06-11

**Authors:** Antonio Vitiello, Jessica Mok, Mohamed Elkalaawy, Andrea Pucci, Andrew Jenkinson, Rachel Battheram, Vincenzo Pilone, Marco Adamo

**Affiliations:** 1 Bariatric Centre for Weight Management and Metabolic Surgery, University College London Hospital (UCLH), London NW1 2BU, UK; 2 Advanced Biomedical Sciences Department, University of Naples Federico II, 80131 Naples, Italy; 3 Public Health Department, University of Naples Federico II, 80131 Naples, Italy

**Keywords:** bariatric surgery, staple line leak, sleeve gastrectomy, staple line reinforcement, bleeding

## Abstract

**Introduction:** Various techniques and reinforcements have been proposed over the years in order to prevent leaks and bleeding after sleeve gastrectomy (LSG). The aim of this study was to retrospectively compare the staple line complication (SLC) rate in patients who underwent LSG with the use of bioabsorbable membrane (GORE^®^ SEAMGUARD^®^, GoR) for staple line versus those who received no reinforcement. **Methods:** Data on all consecutive patients undergoing LSG between 1 January 2014 and 31 December 2018 were retrospectively reviewed. Patients were divided into two groups: the GoR+ group if the SeamGuard (GoR) was used and the GoR− group if no reinforcement was applied on the staple line. Preoperative demographics and rate of SLC were compared between groups. All cases of SLC coming from other centers were also reviewed. **Results:** A total number of 626 LSGs were performed at our institution during the study period. GoR was applied in 450 (71.9%) cases (GoR+ group), while NR was used in 176 (28.1%) patients (GoR− group). Two (1.13%) cases of leaking and two (1.13%) cases of bleeding occurred in the GoR− group, while no SLC was recorded in patients who received GoR (*p* < 0.05). Thirteen cases of SLC coming from other institutions were treated at our hospital; all these cases were performed without any SLR. **Conclusion:** In our case series, the use of GoR reduced the rate of SLC after LSG. In all cases of SLC coming from other institutions, no reinforcement had been applied on the staple line during LSG.

## 1. Introduction

Laparoscopic sleeve gastrectomy (LSG) was initially reported in 1993 by Marceau as the first part of the Duodenal Switch Bilio-Pancreatic Diversion (DS-BPD) in order to avoid pyloric dysfunction through the preservation of the vagal nerves. In 1999, Gagner performed LSG as the first step procedure for the Roux-en-Y Gastric Bypass (RYGB) in patients with BMI > 60 kg/m^2^.

After rapid diffusion worldwide, LSG currently represents the most-performed metabolic procedure since 2014 [1], and the main reasons for its long-lasting success are its laparoscopic feasibility and long-term effectiveness.

The SLEEVEPASS [2] randomized controlled trial demonstrated that, although gastric bypass compared to sleeve gastrectomy was associated with a greater percentage of excess weight loss at 5 years, the difference was not statistically significant.

However, the absence of anastomosis does not mean this procedure is free of staple line complications (SLCs), such as leaking and/or bleeding [3]. Even if SLCs are reportedly rare and may be influenced by surgical expertise [4,5], prevention is still mandatory due to the continuously growing numbers of LSG cases globally [6].

Various techniques and reinforcements have been proposed over the years in order to prevent leaks and bleeding after LSG [7,8]. A recent analysis of the Metabolic and Bariatric Surgery Accreditation Quality Initiative Program (MBSAQIP) database (513,354 LSGs performed between 2015 and 2019) has shown that 25.6% of the cohort had no reinforcement (NR), 54.0% had buttressing (BR), 9.8% had oversewing (OS), and 10.8% had a combination of OS and BR. Interestingly, only BR and OS were both associated with a decreased bleeding rate [9].

In our institution, LSG is performed following a standardized technique by the same surgical team; the main difference was represented by the liberal use of staple line reinforcement.

The aim of this study was to retrospectively compare the SLC rate in patients who underwent LSG with the use of a bioabsorbable membrane (GORE^®^ SEAMGUARD^®^, GoR, Flagstaff, AZ, USA) for staple line versus those who received no reinforcement.

## 2. Materials and Methods

Data on all consecutive patients undergoing LSG under the same surgical team (5 different surgeons) between 1 January 2014 and 31 December 2018 were retrospectively retrieved from a prospective maintained database. Patients were divided into two groups: the GoR+ group if the SeamGuard (GoR) was used and the GoR− group if no reinforcement was applied on the staple line. Preoperative demographics and the rate of SLC were compared between groups.

Our database was further reviewed to find all patients with SLC treated at our institution coming from other hospitals. The original bariatric center was contacted to receive information on SLR.

### 2.1. Surgical Technique

A five-trocar approach (3 × 12 mm, 2 × 5 mm) was used. Gastrectomy started 4 cm from the pylorus over a 36–38 French bougie. SeamGuard was randomly used as reinforcement depending on the surgeon’s preference, but in all cases, the staple line was carefully inspected, and metallic clips were applied in case of bleeding spots. An average of 5 cartridges (green for the antrum, yellow for the body, and blue for the fundus) of a 60 mm stapler were used. The last cartridge was fired 1 cm lateral to the esophagogastric junction. No drain or nasogastric tube was routinely left at the end of the procedure.

### 2.2. Preoperative and Postoperative Care

All patients met internationally accepted criteria for Metabolic Bariatric Surgery (MBS). Every patient underwent a first clinic appointment with the surgeon, the psychologist, and the dietitian before surgery. A multidisciplinary team also including the endocrinologists met every week to discuss each patient. Individuals undergoing bariatric surgery were admitted and gave consent on the morning of the procedure.

After surgery, patients were moved from the operating room to the post anesthesia care unit (PACU) to check vitals in the first postoperative hours. Then, patients were admitted to a ward and cared for by nurses and reviewed daily by doctors. Blood tests were performed 6 h after surgery and then daily.

A liquid diet was started the day of surgery, and asymptomatic patients were discharged on postoperative day 1–2. In cases of fever and/or tachycardia, a leak was suspected and a blood test was required to check inflammatory indexes. In cases of leukocytosis and/or high CRP, an abdominal CT was performed.

In cases of tachycardia and hypotension, bleeding was suspected and a blood test was required to check hemoglobin. In cases of mild anemia with a stable patient, another blood count was required after 6 h, while in cases of severe hemoglobin drop, an abdominal CT was prescribed to find possible collections.

In all cases of unstable patients, an emergency laparoscopy was performed.

### 2.3. Statistical Analysis

Pearson’s χ^2^ test and *t* tests were, respectively, used for categorical and normally distributed continuous variables. Continuous variables were reported as mean ± standard deviation and categorical variables as percentage. All statistical analyses were performed with Microsoft Excel (Office 365 Version). A *p* value < 0.05 was considered statistically significant.

## 3. Results

A total of 626 LSGs were performed at our institution during the study period. Data on intraoperative use of GoR and postoperative rate of SLC were available for all cases. GoR was applied in 450 (71.9%) cases (GoR+ group), while NR was used in 176 (28.1%) patients (GoR− group). The distribution of GoR+ and GoR− cases among the five surgeons is shown in Table 1. Comparison of preoperative demographics showed that weight and percentage of patients with BMI > 50 were significantly higher in the GoR− group (Table 2).

Two (1.13%) cases of leaking and two (1.13%) cases of bleeding occurred in the GoR− group, while no SLC was recorded in patients who received GoR (Table 3). Bleedings were treated with blood transfusions, while leaks occurred in the upper part of the staple line.

**Figure 1:**
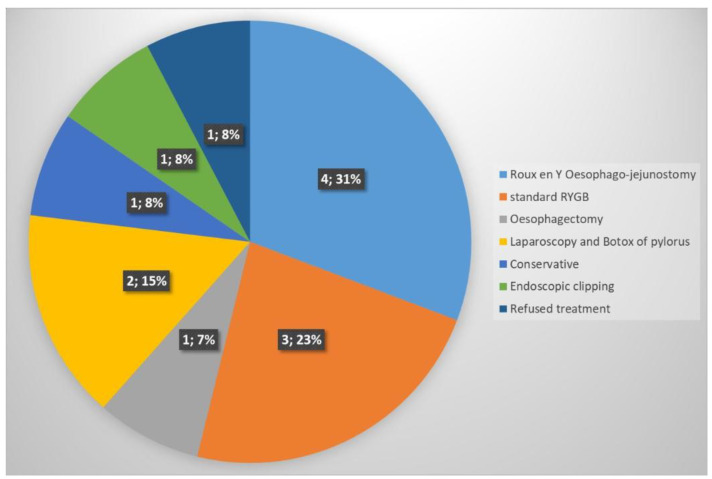
Number and treatments of staple line leaks coming from other hospitals.

Thirteen cases of SLC coming from other institutions were treated at our hospital (Figure 1). All these cases were performed without any SLR (Table 4).

## 4. Discussion

LSG is probably the surgical laparoscopic intervention with the longest staple line. Considering also the high intraluminal pressure, SLCs are difficult to prevent. Reported leak rates are between 0% and 8.0%, and bleeding rates are between 0% and 3% [10].

In 2009, an initial review [11] concluded that there was no reason to believe, at that point, that a reduction in the leak rate occurred due to the use of reinforcement. Indeed, the leak rate has always been small after LSG; thus, the routine reinforcement of the staple line has been questioned. However, a decrease in hemorrhage was demonstrated also in the first reports.

At the Second International Consensus Summit for Sleeve Gastrectomy, the staple line was reinforced by 65.1% of the responders; of these, 50.9% preferred oversewing, 42.1% preferred buttressing, and 7% did both [12]. At the Fifth Summit, the majority of surgeons (43.2%) preferred buttressing material for staple line reinforcement, whereas 28.8% preferred oversewing and 28.0% preferred no staple line reinforcement [13]. In 2012, a first meta-analysis showed that reinforcing the staple line decreased the incidence of postoperative leak and overall complications [14]. In 2013, a second paper confirmed these outcomes in favor of the use of SLR [15]. The following year, a systematic review of 88 included studies representing 8920 patients found that the leak rate in LSG was significantly lower using absorbable polymer membrane (APM) staple line reinforcement than oversewing, nonabsorbable bovine pericardial strips (BPSs), or no SLR [16]. In contrast, in 2016, another paper still confirmed the different effectiveness of different reinforcements, but BPS resulted in the most favorable outcomes [17]. However, a first analysis of randomized controlled trials showed that even if SLR could reduce hemorrhage and overall complications, no obvious advantages of oversewing the staple line were found, and it took longer operative time. Interestingly, no significant reduction in leak rate was evidenced after reinforcement [18].

Interestingly, the impact of the surgical technique used has also been widely evaluated throughout the years. Ten years ago, a first meta-analysis of 9991 LSG patients concluded that bougie size ≥40, but not buttressing or distance from the pylorus, could impact SLC [19]. In 2016 [20], a review confirmed these findings, but recent evidence claims that a bougie size between 33 and 36 may reduce the risk of leaking [21]. Outcomes from the MBSAQIP conversely suggest that bougie is not associated with any change in postoperative leak rates [22].

In our experience, we compared results of different surgeons using the same technique with a standard bougie size of 32–36 Fr and distance from the pylorus of >4 cm. Although different expertise and proficiency may always influence outcomes, due to this standardization, SLCs were likely related to the type of SLR.

Another analysis from the MBSAQIP [9] indeed showed that both SLR and oversewing decreased the rate of bleeding but not of leaking, while another study from the same database concluded that, also in patients on anticoagulation therapy, oversewing prolongs operative time without benefits in terms of SLC [23]. In contrast, a recent meta-analysis demonstrated that only oversewing was an effective method to reduce bleeding and SLC after LSG [24]. Recent studies have confirmed that both SeamGuard and oversewing could reduce SLC after LSG [25,26,27]. Although the use of APM has reduced in recent years [28], in our institutions, we have continued to use GoR as the only SLR in the period of this study. GoR is a synthetic buttressing absorbable material engineered to reduce perioperative leaks and bleeding in a variety of minimally invasive surgeries. A study already demonstrated that the use of GoR significantly reduced the need for the intraoperative use of hemostatic clips on the staple line. A more recent article demonstrated that the use of GoR on the whole staple line reduces the postoperative bleeding rate and hospital stay. Our data certainly confirm that no reinforcement is associated with a higher risk of SLC. Even if we do not have data from the original centers, it is noteworthy that all patients coming from other hospitals were cases with no reinforcement. Moreover, in our experience, SLC only occurred when GoR was not used, while a 0% rate was observed in the GoR+ group.

### Strengths and Limitations

It is arguable that in the GoR− group, patients had higher preoperative weight due to the higher rate of patience with BMI > 50. It is well-known that these individuals may have a higher risk of postoperative complications. However, groups were comparable for mean BMI and other demographics.

More than a comparison between techniques, our research may be seen as a comparison between surgeons since the same surgeons used the same reinforcement or no reinforcement.

However, as explained, the most important strength of this paper is that we presented a large sample (>600 cases) over a five-year period of five different expert surgeons using a standardized technique. Due to this standardization, the SLCs were likely related to the type of SLR.

## 5. Conclusions

In our experience, the no reinforcement technique was associated with a higher risk of staple line complications. The rate of SLCs after GoR use was 0%, while in the GoR− group, there was a significantly higher percentage of leaking and bleeding.

## Figures and Tables

**Table 1:** Distribution of SeamGuard (SG) use among surgeons in our institution.

	Surgeon 1	Surgeon 2	Surgeon 3	Surgeon 4	Surgeon 5
**GoR+** *(n = 450)*	216	119	115	0	0
**GoR−** *(n = 176)*	0	0	0	34	142

**Table 2:** Preoperative demographics.

	Group GoR+ *(n = 450)*	Group GoR− *(n = 176)*	*p* Value
Age (years)	41.5 ± 11.6	43.1 ± 9.9	*0.09*
BMI (Kg/m^2^)	44.2 ± 7.6	45.1 ± 6.5	*0.13*
Preoperative weight (Kg)	123.7 ± 16.4	127.8 ± 17	***0.006***
Sex (F/M)	338/112	132/44	*0.9*
Previous abdominal surgery (YES/NO)	139/311	66/110	*0.11*
Patients with BMI > 50	31/399	51/145	***0.03***

**Table 3:** Comparison of staple line complications between the two groups.

	Group GoR+ *(n = 450*)	Group GoR− *(n = 176)*	*p* Value
Staple line leak	0	2 (1.13%)	***0.02***
Staple line bleeding	0	2 (1.13%)	***0.02***
Total staple line complications	0	4 (2.26%)	***0.001***

**Table 4:** Staple line complications coming from other centers.

	Reinforcement	No Reinforcement
Staple line leak	0	11
Staple line bleed	0	2
Total staple line complications	0	13

## Data Availability

Data are unavailable due to privacy.
